# Evaluating the Combined Effects of Erythromycin and Levofloxacin on the Growth of *Navicula* sp. and Understanding the Underlying Mechanisms

**DOI:** 10.3390/plants12132547

**Published:** 2023-07-04

**Authors:** Jie Yang, Waqas Ahmed, Sajid Mehmood, Wenjie Ou, Jiannan Li, Wenxin Xu, Lu Wang, Mohsin Mahmood, Weidong Li

**Affiliations:** 1 Key Laboratory of Agro-Forestry Environmental Processes and Ecological Regulation of Hainan Province, Hainan University, Haikou 570228, China; yangjie8681@yeah.net (J.Y.);; 2 Center for Eco-Environment Restoration Engineering of Hainan Province, Hainan University, Haikou 570228, China; 3 Collaborative Innovation Center of Ecological Civilization, Hainan University, Haikou 570228, China

**Keywords:** *Navicula* sp., antibiotic pollutants, aquatic ecosystems, toxic effects, primary producers

## Abstract

*Navicula* sp., a type of benthic diatom, plays a crucial role in the carbon cycle as a widely distributed algae in water bodies, making it an essential primary producer in the context of global carbon neutrality. However, using erythromycin (ERY) and levofloxacin (LEV) in medicine, livestock, and aquaculture has introduced a new class of pollutants known as antibiotic pollutants, which pose potential threats to human and animal health. This study aimed to investigate the toxic effects of ERY and LEV, individually or in combination, on the growth, antioxidant system, chlorophyll synthesis, and various cell osmotic pressure indexes (such as soluble protein, proline, and betaine) of *Navicula* sp. The results indicated that ERY (1 mg/L), LEV (320 mg/L), and their combined effects could inhibit the growth of *Navicula* sp. Interestingly, the combination of these two drugs exhibited a time-dependent effect on the chlorophyll synthesis of *Navicula* sp., with ERY inhibiting the process while LEV promoted it. Furthermore, after 96 h of exposure to the drugs, the activities of GSH-Px, POD, CAT, and the contents of MDA, proline, and betaine increased. Conversely, the actions of GST and the contents of GSH and soluble protein decreased in the ERY group. In the LEV group, the activities of POD and CAT and the contents of GSH, MDA, proline, and betaine increased, while the contents of soluble protein decreased. Conversely, the mixed group exhibited increased POD activity and contents of GSH, MDA, proline, betaine, and soluble protein. These findings suggest that antibiotics found in pharmaceutical and personal care products (PPCPs) can harm primary marine benthic eukaryotes. The findings from the research on the possible hazards linked to antibiotic medications in aquatic ecosystems offer valuable knowledge for ensuring the safe application of these drugs in environmental contexts.

## 1. Introduction

Antibiotics, classified as pharmaceutical and personal care products (PPCPs), are emerging pollutants, extensively utilized in medicine, industry, animal husbandry, aquaculture, and various other domains [1]. Erythromycin (ERY), a broad-spectrum macrolide antibiotic (ATB), and levofloxacin (LEV), a third-generation fluoroquinolone (FQ), have gained widespread usage in the treatment of human and veterinary diseases [2]. But, due to the poor removal effect of traditional sewage treatment technology, they are widespread in the aquatic environment [3]. In fact, high concentrations of ERY have been recorded in effluents (6000 ng/L) and surface waters (1700 ng/L), reaching levels of mg/L [4]. The long-term existence of antibiotics and their degradation products in water can induce drug-resistant bacteria, impacting the microbial community and its food chain and damaging the ecological balance [5]. The presence of drugs in the aquatic environment is a significant concern as they can accumulate within organisms through the food chain, potentially leading to adverse effects on human health [6]. As primary producers in aquatic ecosystems, microalgae exhibit high sensitivity to the stress caused by pollutants and are considered one of the most vulnerable organisms to the impacts of antibiotics. The study’s findings revealed that antibiotics exerted ecotoxic effects on microalgae, including the induction of oxidative stress, chlorophyll synthesis disruption, and microalgae growth inhibition [7]. In addition, studies have shown that LEV at 320 mg/L can significantly inhibit the algae’s growth, reducing the carbohydrate and lipid content in the cell [8].

Diatoms are primary producers in aquatic ecosystems. They have a wide distribution in various water bodies, including seawater, rivers, lakes, reservoirs, wetlands, and mangroves. Currently, there are approximately 100,000 known species of diatoms worldwide [9]. Studies have shown that diatoms can produce up to 4.5–5.0 Gt of organic carbon through photosynthesis yearly, a very important biological energy and primary productivity in the ocean and freshwater [10]. Especially in the ocean, entering the ocean accounts for about 40% of the primary productivity. Its role in the global carbon cycle can be comparable to that of terrestrial rainforests. At the same time, it is one of the main foods for fish, shellfish, and other aquatic animals. It is worth emphasizing that algae are widely used in biological monitoring, paleoecological reconstruction, and stratigraphic relations [11,12].

*Navicula* sp. (hereafter referred to as *Navicula*) is a type of diatoms. It is usually applied as an environmental indicator in freshwater [13]. However, it is also a widely distributed marine diatom [14]. As far as we know, there are relatively few studies on the joint compared to the single toxicity of antibiotics to algae. Nevertheless, all kinds of antibiotics can coexist in the natural environment [15], and the toxic effects of mixtures of antibiotics are more complicated than those of individual antibiotics in most cases rather than being simply additive [16]. Although the combined toxicity of antibiotic mixtures on algae growth has been explored in several studies, the underlying mechanisms responsible for this phenomenon remain relatively unexplored. These mechanisms likely involve vital physiological functions of algae, such as antioxidant defense systems, metabolic transformations, and photosynthesis. Gaining a comprehensive understanding of these mechanisms is crucial for assessing the combined toxicity of antibiotics and their potential environmental risks. With reference to the works of Wang et al. [17] and Xiong et al. [18], we have chosen to investigate the effects of 1mg/L ERY and 320 mg/L LEV on *Navicula*, as these concentrations were deemed appropriate for our study. The objectives of this study were: (1) to explore the stress and toxicological effects of single and combined antibiotics (ERY and LEV) on *Navicula* under optimal growth conditions and (2) to specifically focus on the impact of single and combined antibiotic treatments on the growth, chlorophyll synthesis, and antioxidant system of *Navicula*.

## 2. Results

### 2.1. Influence of ERY and LEV on the Growth of Navicula

**Figure 1:**
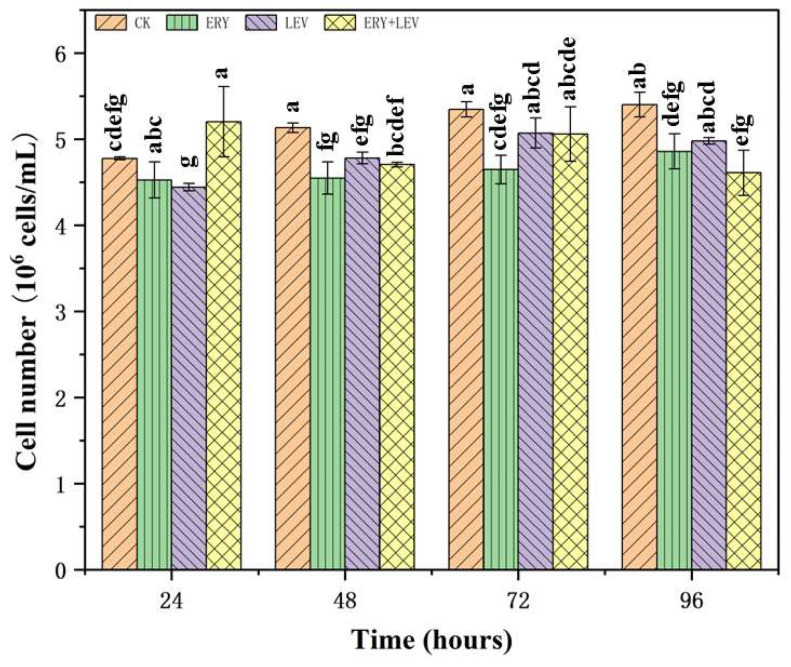
Effect on cell number of *Navicula*. Different letters indicate significant differences (*p* < 0.05). Bars means ± SD (*n* = 3).

The initial culture density of *Navicula* was 4.7 × 10^6^ cells/mL. After 96 h of drug exposure, the results showed that ERY, LEV, and their combined effects had significant inhibitory effects on the growth of *Navicula* compared with the control group (Figure 1). The cell density of different experimental groups (G1, G2, G3) was decreased. They were 0.90 times, 0.92 times and 0.85 times lower than the control group, respectively. Among them, G2 has the highest cell density and G3 has the lowest cell density. The combined effect was the most toxic. During 96 h of treatment, the algal cells of G1 (1 mg/L ERY) and G2 (320 mg/L LEV) showed a continuous inhibition trend compared with the control group. However, compared with the control group, the algal cells of G3 (1 mg/L ERY +320 mg/L LEV) added with compound drugs increased significantly at the treatment time of 24 h, reaching a peak of 5.2 × 10^6^ cells/mL but subsequently showed a downward trend as the G1 (1 mg/L ERY) and G2 (320 mg/L LEV).

### 2.2. Photosynthetic Pigment Content of Navicula

**Figure 2:**
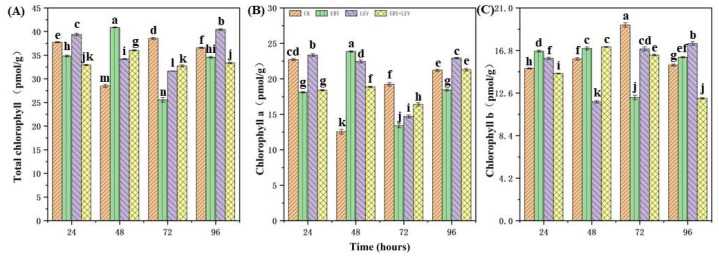
Photosynthetic pigment content in *Navicula* after 96 h of exposure. (**A**) Total chlorophyll, (**B**) Chlorophyll a, and (**C**) Chlorophyll b. Different letters indicate significant differences (*p* < 0.05). Bars are means ± SD (*n* = 3).

The change in the photosynthetic pigment content of *Navicula* is shown in Figure 2. The content of chlorophyll a and total chlorophyll in G1 *Navicula* increased significantly at 48 h and then decreased. At the same time, the content of chlorophyll b decreased significantly after 72 h of treatment, and then increased close to the CK. Chlorophyll a content of G2 *Navicula* increased significantly at 24 h, while chlorophyll b content and total chlorophyll content increased significantly at 96 h. However, the content of chlorophyll b of G3 *Navicula* increased after 48 h of treatment, and there was no significant difference between the total chlorophyll content and the chlorophyll a content.

### 2.3. The Antioxidant System of Navicula

**Figure 3:**
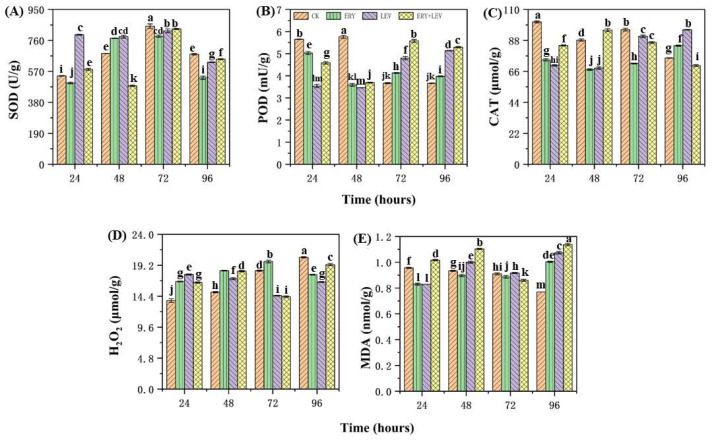
Changes in the activities of antioxidant enzyme and the contents of H_2_O_2_ and MAD in *Navicula* after 96 h of exposure. (**A**) The activity of superoxide dismutase (SOD); (**B**) the activity of peroxidase (POD); (**C**) the activity of catalase (CAT); (**D**) the content of hydrogen peroxide (H_2_O_2_); (**E**) the content of malondialdehyde (MDA). Different letters indicate significant differences (*p* < 0.05). Bars are means ± SD (*n* = 3).

The activity changes of superoxide dismutase (SOD), peroxidase (POD), and catalase (CAT) of *Navicula* and the content changes of hydrogen peroxide (H_2_O_2_) and malondialdehyde (MDA) are shown in Figure 3. After 96 h of treatment with ERY, LEV, and their combination, overall, the SOD activity of the *Navicula* was lower than the CK, and the lowest SOD activity was 481.46 U/g in the G1 group (Figure 3A). After 96 h, the POD activity of the *Navicula* in G1, G2, and G3 was higher by 1.09, 1.40 and 1.45 times, compared with the CK, respectively. G3 showed the highest POD activity at 5.3 mU/g (Figure 3B). In addition, after 96 h, the CAT activity in G3 was the lowest at 70.18 μmol/g, whereas it increased in G1 and G2 increased by 1.12 and 1.27, compared with the CK, respectively, while the CAT activity in the G2 was the highest at 95.31 μmol/g (Figure 3C). Meanwhile, the content of H_2_O_2_ in the CK increased gradually with the incubation time, while it was lower than that of the CK after 96 h of drug exposure (Figure 3D). Contrarily, the content of MDA in the CK decreased gradually with the incubation time. However, the content of MDA in *Navicula* in G1, G2, and G3 increased first, then decreased, and then increased with the time of drug exposure. It was increased by 1.30, 1.39, and 1.48 times, respectively, compared with the CK after 96 h of drug exposure (Figure 3E).

**Figure 4:**
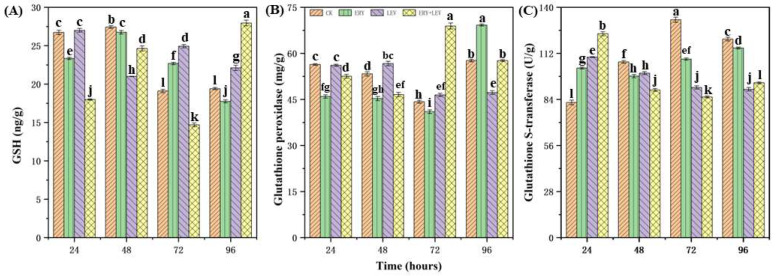
Effects on (**A**) glutathione (GSH), (**B**) glutathione S-transferase (GSH-Px), and (**C**) glutathione peroxidase (GST) of *Navicula*. Different letters indicate significant differences (*p* < 0.05). Bars are means ± SD (*n* = 3).

After 24 h of drug exposure, the glutathione S-transferase (GSH-Px) activity in G1, G2, and G3 was 1.25, 1.36, and 1.51 times higher than that of the CK (Figure 4B). After 72 h, the glutathione peroxidase (GST) activity of *Navicula* was significantly higher by 1.56 times, compared with the CK (Figure 4C). After 96 h, the GST activity in G1 was significantly higher than that in the control group, which was 69.23 ng/g, 1.20 times higher than that in the control group. The GST activity of the scaphoid in G2 was 47.23 ng/g, lower than that of the CK. However, there was no significant change observed in G3. The activity of GSH-Px in the scaphoid of G1, G2, and G3 was 115.29 U/g, 90.20 U/g, and 94.10 U/g, respectively, which was lower than the CK (Figure 4A). The glutathione (GSH) content in G1 was then lower than that in the control group. Nevertheless, the GSH content in G2 and G3 was 1.14 times and 1.44 times higher than that in the control group, respectively (Figure 4A).

### 2.4. Partial Osmotic Substances of Navicula

**Figure 5:**
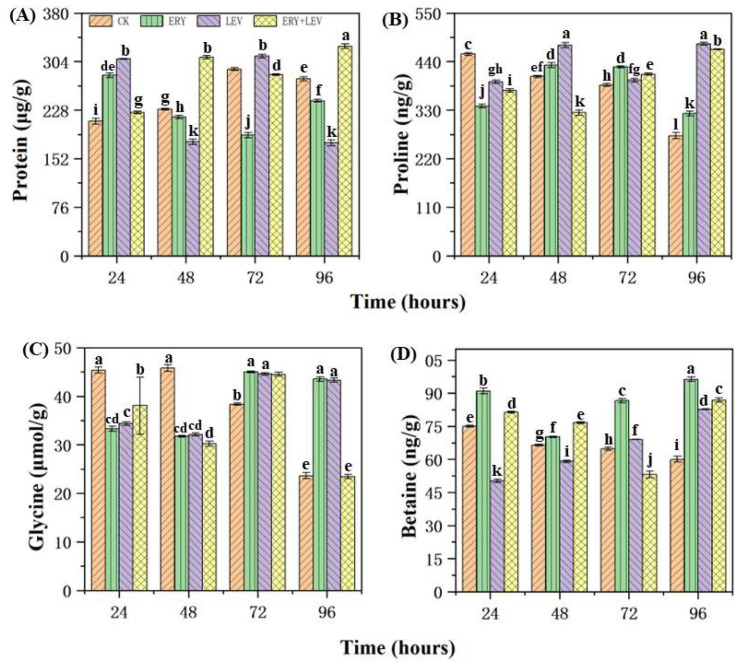
Effects on (**A**) protein, (**B**) proline, (**C**) glycine, and (**D**) betaine of *Navicula*. Different letters indicate significant differences (*p* < 0.05). Bars are means ± SD (*n* = 3).

The contents of soluble protein, proline, glycine, and betaine in *Navicula* exposed to drugs are shown in Figure 5. Firstly, at the 24-h stage, the soluble protein content of *Navicula* in G1, G2, and G3 was 1.34, 1.46 and 1.07 times higher than the CK, respectively (Figure 5A). G1 and G2 were more evident than G3. After 48 h, the soluble protein content of *Navicula* in G3 was significantly higher than the CK, which was 1.35 times the CK, while G1 and G2 were lower than the CK, respectively. At the 72 h of drug exposure, the soluble protein content of *Navicula* in G1 and G3 was 188.82 μg/g, lower than that in the CK, while in G2, it was higher than that of the CK (312.51 μg/g). After 96 h of drug exposure, G3 was significantly higher than the CK, which was 327.96 μg/g, while the soluble protein content of *Navicula* in G1 and G2 was lower than the CK, which is 242.73 μg/g and 176.78 μg/g, respectively. Secondly, the content of proline in the CK decreased gradually with the culture time (Figure 5B). At 24 h, G1, G2, and G3 showed a lower proline content than the CK. However, with the increase in exposure time, the proline content in G1 and G2 was higher than in the control group. Especially when the drug was exposed for 48 h, the proline content in G2 was significantly higher than that in the control group, which was 476.99 ng/g. After 96 h of drug exposure, the proline content in G1, G2, and G3 was 1.18, 1.76, and 1.71 times higher than the CK. The glycine contents in *Navicula* exposed to drugs are then shown in Figure 5C. The glycine content in the control group decreased gradually with the culture time. At the 24 and 48 h of drug exposure, the glycine content of *Navicula* in G1, G2, and G3 was lower than the CK. However, after 72 h of drug exposure, the glycine content of *Navicula* in G1, G2, and G3 was higher than the CK and reached the maximum value of 45.03 μmol/g, 44.59 μmol/g, and 44.54 μmol/g. However, after 96 h of drug exposure, the glycine content in G1 and G2 was higher than that in the control group, and 1.84 and 1.83 times higher than that of the CK, respectively. There was no significant difference between G3 and the control group. Finally, for betaine (Figure 5D), the content in the control group decreased gradually with the culture time. With the increase in the drug exposure time, the betaine content in G1 was always higher than in the CK. The betaine content in G2 was lower than that in the control group at 24 and 48 h of drug exposure, but with the increase in exposure time, it was higher than that in the control group after 72 h. The betaine content of *Navicula* in G3 was lower than that of the CK at 72 h of drug exposure. In general, after 96 h of drug exposure, the betaine content of *Navicula* in G1, G2, and G3 was 1.61, 1.38, and 1.48 times higher than that of the CK, respectively.

## 3. Materials and Methods

### 3.1. Chemicals and Reagents

Erythromycin (ERY, CAS 114-07-8, C_37_H_67_NO_13_, with a purity of greater than 98%) was purchased from the Shanghai Macklin Biochemical Co., Ltd. (Shanghai, China). Levofloxacin (LEV, CAS 100986-85-4, C_18_H_20_FN_3_O_4_, with a purity greater than 98%) was purchased from the Hainan Zhengheng Technology Co., Ltd. (Haikou, China). The physicochemical properties of different chemicals used in this study are presented in Table 1.

### 3.2. Microalgal Pre-Culture

The *Navicula* sp. (MASCC-0035) was purchased from the microalgae germplasm bank of the Institute of Oceanography, Chinese Academy of Sciences (Qingdao, China). The microalgal strain was pre-cultured in an F/2 medium [19,20] at 24 ± 1 °C in an artificial incubator under illumination at 2000 Lx under a 12 h/12 h light/dark cycle. Algal cells in the exponential growth phase were used for the following experiments. Cultures were shaken by hand three times a day to circumvent the adhesion of microalgae cells onto the wall of the glass beaker.

### 3.3. Procedures for the Exposure Experiments

The exposure experiment was conducted in a series of 1 L glass beakers with F/2 medium. The experiment was divided into three groups. (1) G1: ERY powder was added to the F/2 medium to make its concentration reach 1 mg/L; (2) G2: we added LEV powder in F/2 medium to make its concentration reach 320 mg/L; (3) G3: ERY and LEV powder were added to the F/2 medium to make the ERY concentration reach 1 mg/L and LEV concentration reach 320 mg/L. Pure culture without drugs served as the control group. The glass beakers were shaken five times a day during culture to ensure the best pattern of *Navicula* growth. Three replicates were set per group. Algae were cultivated under the same conditions as applied in pre-acculturation for 4 days. After 0, 24, 48, 72, and 96 h of cultivation, the cultures were collected to measure physiological and biochemical indices.

### 3.4. Measurement of Cell Density-Spectrophotometry

Firstly, the high-density algal fluid of *Navicula* was diluted with the culture medium into different density gradients. A hemocytometer (22 × 26 × 0.5 mm) was then used under an optical microscope to count the density of algal cells in each density gradient solution (as an average of three counts). The absorbance of the algal solution was measured with an ultraviolet-visible spectrophotometer at 680 nm, and the linear relationship between algal cell numbers and OD_680_ was obtained after extensive data analysis and was calculated as follows:

$$Cell\;numbers\;of\;Navicula\;\mathrm{sp}.\;(\mathrm{cells}\cdot {\mathrm{mL}}^{-1})=8\times 10^{6}{OD}_{680}-10^{7}(R^{2}=0.9973)$$

### 3.5. Chlorophyll Determination

Chlorophyll was measured according to the methodology of Zhang et al. [21]. *Navicula* with a fresh weight of about 1g was ground into a powder with liquid nitrogen, dissolved in nine volumes of phosphate-buffered saline (pH 7.4) in an ice bath and centrifuged (15 min at 5000 rpm). The contents of total chlorophyll, chlorophyll a, and chlorophyll b in the supernatant were detected according to the instructions of the kits. Kits were provided by the Jiangsu Kete Biotechnology Co., Ltd., Yancheng, China. Three biological replicates were evaluated for each sample.

### 3.6. Determination of Antioxidant Activities

To further understand the mechanism underlying the toxicity of ERY and LEV toward *Navicula*, the effects of these two antibiotics on the antioxidant defense system of this microalga were evaluated by the method, as described by Jiao et al. [22] and Sun et al. [23]. The levels of hydrogen peroxide (H_2_O_2_), malondialdehyde (MDA), and glutathione (GSH) and the activities of catalase (CAT), peroxidase (POD), glutathione peroxidase (GST), glutathione S-transferase (GSH-Px), and superoxide dismutase (SOD) were determined using commercial assay kits purchased from the Jiangsu Kete Biotechnology Co., Ltd. (Yancheng, China).

### 3.7. Determination of Osmoregulation Substances

To further understand the potential mechanism of the effects of ERY and LEV on the growth of this microalgae, the effects of these two antibiotics on the osmoregulating substances (soluble protein, glycine, betaine, and proline) of the microalgae were evaluated. Algae were collected 24, 48, 72, and 96 h after treatment. Each group was repeated three times. The crude extract of algae liquid is prepared according to León-Vaz [24]. Subsequently, according to the manufacturer’s instructions, we used a commercial kit (Jiangsu Kete Biotechnology Co., Ltd., Yancheng, China) to evaluate the osmotic adjustment substances of *Navicula.*

### 3.8. Statistical Analyses

The collected data were analyzed using Statistica 8.1. To ensure the validity of the analysis, we initially conducted tests to assess the homogeneity of variance and checked for data normality using the Shapiro–Wilk test. The results confirmed that the data followed a normal distribution and exhibited skewness and kurtosis within the standard range. Subsequently, a one-way analysis of variance (ANOVA) was performed to conduct further data analysis, followed by post hoc tests. The results were presented as means ± standard deviation (SD) in the figures. The statistical analysis involved ANOVA, followed by Tukey’s multiple comparison test, with a significance level set at *p* < 0.05 to determine the statistical significance.

## 4. Discussion

### 4.1. Effect on the Growth of Navicula

Microalgae are at the bottom of aquatic ecosystems’ food chain and are critical organisms for monitoring water quality [25]. As a traditional toxicological evaluation index, the growth rate of algal cells can intuitively express the stress effect of exogenous pollutants on algal growth [26]. The results showed that both ERY and LEV could inhibit the growth of *Navicula* sp, which may be related to the toxic effect of antibiotics on microalgae cells. This is consistent with the findings of Yang et al. [8] and Li et al. [27]. Additionally, it is possible that at specific concentrations, these antibiotics exert a toxic “excitation” effect, leading to an increase in microalgae biomass [28]. The “excitation” effect of *Navicula* on the stimulation of mixed drugs promoted an increase in algal cells, which could be responsible for the increase in algal cell numbers after 24 h of treatment with G3. The increased toxicity and inhibition can be attributed to the cell function disorder caused by lipid peroxidation. For example, antibiotic stress induces lipid peroxidation of cell membranes and organelle membranes, which in turn affects chlorophyll synthesis occurring on thylakoid membranes [29,30]. In addition, antibiotic stress may inhibit the biosynthesis and gene expression of enzymes related to DNA replication, directly inhibiting microalgae growth [31,32]. This is why the combination of ERY and LEV was the most toxic after 96 h of exposure.

### 4.2. Effects on the Photosynthetic Pigment Content of Navicula

Photosynthesis is closely related to the growth and metabolism of microalgae [33]. Chlorophyll plays a vital role in light energy absorption, transmission, and conversion, especially for autotrophs such as algae. The change in its content will directly affect the normal photosynthesis of algae, and then affect the synthesis of other organic substances (such as various proteins) in algae cells, thus reflecting the advantages and disadvantages of biological growth and development at each stage [34]. The results of G1 and G2 showed that ERY and LEV could inhibit the photosynthesis of *Navicula*, which led to the inability of algae cells to provide enough energy for their growth, thus reducing the growth rate of algae cells [35]. On the other hand, the concentration of the drug can affect the synthesis of chlorophyll a in photosynthesis with the increase in exposure time and then maintain the stability of photosynthesis by stimulating algal cells to produce more chlorophyll a, to resist the influence of an external adverse environment [36]. Some studies have shown that ERY inhibits the chlorophyll biosynthesis of algae, and thylakoid may be the target site of ERY [37]. If the content of chlorophyll decreases, the photosynthesis intensity, the metabolism, the cell’s reproductive capacity, and the number of algal cells also decrease. This is a manifestation of algal cells in the presence of antibiotics and a physiological adaptation of algae. The experiment showed that 1 mg/L ERY has a more prominent effect on the photosynthesis of *Navicula* than 320 mg/L LEV. After 96 h of mixed exposure to the two drugs, the chlorophyll had no significant change compared with the control group. This may be because ERY inhibits the synthesis of chlorophyll in *Navicula*. However, LEV still can stimulate the photosynthesis of algae cells, enabling them to provide energy for their growth to resist the impact of an adverse external environment. They may show strong antagonism [38].

Glycine is mainly abundant in chloroplasts and plays an important role in the regulation and protection of thylakoid membranes, thus maintaining photosynthetic efficiency. Glycine has a unique role in promoting plant growth, especially photosynthesis. It can increase plant chlorophyll’s content, improve enzyme activity, promote carbon dioxide penetration, and make photosynthesis more vigorous [39]. With the increase in drug exposure time, the glycine content in *Navicula* reached the peak at 72 h, which was higher than that in the control group. After the glycine content in the algae increases, the photosynthesis of plants is promoted. Therefore, after 96 h of drug exposure, the chlorophyll in the algae is increased compared with 72 h, providing energy for maintaining the growth of algae cells.

### 4.3. Effects on the Antioxidant Responses of Navicula

When organic pollutants are transformed in organisms, they generate redox cycles, a large number of active oxygen, and induce the activity of enzymes in the antioxidant defense system [40]. SOD and GSH show a good correlation after animals, some higher plants, algae, and other organisms are exposed to organic pollutants [41]. Therefore, the activities of some enzymes in the antioxidant defense system can be used as biomarkers of the protective effects of organic pollutants, including antibiotics.

The oxidative stress induced by PPCPs on algal cells is one of the important mechanisms of its toxicity to microalgae. Microalgae cells exposed to PPCPs will suffer oxidative stress and ROS, which will affect the synthesis of biological molecules (such as proteins, pigments, and lipids) [42]. In addition, ROS will peroxide fatty acids to form MDA. MDA is the final decomposition product of the lipid peroxidation reaction. It combines with protein to cause cross-linking within and between protein molecules. The synthesis of protein is blocked. It indicates cell peroxidation [43]. The increase in its content indicates that the body is under oxidative stress, so it is usually used to show the degree of oxidative damage to cells after external stimulation [30]. The increase in MDA content is a universal sign of oxidative stress in microalgae exposed to PPCPs. When microalgae were exposed to drugs for 96 h, the MDA content of all treatments was higher than that of the control group. The experimental results showed that the algal cells were oxidized to different degrees when the *Navicula* were exposed to the drug. The damage to algal cells in the mixed drug was more than in the single drug.

The oxidative stress reaction in algal cells will affect the production of antioxidant enzymes, such as SOD, CAT, and POD, which is the protective mechanism of microalgae against ROS [44]. When *Navicula* are exposed to drugs, their cells produce many reactive oxygen species due to stress. To eliminate redundant reactive oxygen species, the SOD activity as the first line of defense will first increase [45,46]. In addition, SOD in algal cells undergoes enzymatic reactions to produce H_2_O_2_. With the increase in H_2_O_2_ content in algal cells, the toxicity to algal cells was gradually enhanced. In order to resist the toxicity of H_2_O_2_, POD and CAT activities were increased in the peroxisome of *Navicular* cells. POD and CAT in microalgae can degrade the H_2_O_2_ produced by SOD disproportionation into nontoxic water and oxygen, eliminating the toxicity of hydrogen peroxide to the body [47].

In addition to the above antioxidant enzymes, GSH, GSH-Px and GST can also protect plants from pollutants and ROS damage. GSH is a tripeptide composed of glutamic acid, cysteine, and glycine, containing the sulfhydryl group, which has an antioxidant and integrated detoxification effect. On the one hand, GSH can directly eliminate ROS. On the other hand, GSH can also remove environmental xenobiotics or ROS by participating in the ascorbic acid glutathione cycle or combining with electrophilic groups as a synergistic substrate in removing electrophilic environmental xenobiotics [48,49]. GST is the key enzyme of the GSH binding reaction, which can catalyze the binding response of nucleophilic glutathione with various electrophilic foreign chemicals [50]. Many exogenous chemicals easily form some bioactive intermediates in the first phase reaction of biotransformation. They can covalently combine with important components of cellular biological macromolecules, causing damage to the body. After GSH is combined with glutathione, such a covalent combination can be prevented, and detoxification can be achieved. When the *Navicula* are exposed to the drug for 24 h, the activity of GST in the algal cells is significantly enhanced, which catalyzes the binding reaction between GSH and foreign pollutants, to protect it from damage caused by the toxicity of intermediate products [51]. However, with the increase in exposure time, the activity of GST gradually decreased, indicating that the exogenous pollutants have an inhibitory effect on the activity of GST. At the same time, the algal cells were also damaged. GSH-Px is ubiquitous in plants. Its expression is induced by many environmental factors and plays an important role in plant growth and development, secondary metabolism, and stress resistance [52].

### 4.4. Effects of Partial Osmotic Substances of Navicula

Betaine, proline, and protein are fundamental indicators in plant cells, closely related to cell osmotic pressure. Betaine is a kind of non-toxic small molecule osmotic regulation substance that can quickly penetrate plant organs and improve the ability of cells to absorb or retain water. Chemically it is a quaternary ammonium-type water soluble alkaloid [53]. Proline, a free amino acid, is not only an ideal osmotic regulator, but also a protective substance for membranes and enzymes and a free radical scavenger to protect plant growth under osmotic stress [54]. After 96 h of exposure to the drug, the contents of betaine and proline were higher than those of the control group. This may be because betaine can enhance the resistance to the adverse environment by increasing the contents of proline, soluble protein, and other osmotic regulatory substances in plants under stress. Its high concentration does not affect many enzymes and other biological macromolecules and even has a protective effect [55]. At the same time, some studies have found that betaine can improve enzyme activity by indirectly increasing the expression of genes related to the antioxidant enzyme system [56]. Therefore, the increase in betaine content also provides some help for antioxidant enzymes to resist external toxic stress. The role of proline in plant oxidative stress has been widely demonstrated in the application experiments of exogenous proline [57] or in the genetic engineering experiments [58] of proline synthesis or degradation. The ability of proline to protect against ROS damage may also explain its role as an antioxidant in alleviating stress-induced oxidative damage. In addition to its direct effect, proline also indirectly clears ROS by enhancing the antioxidant defense system of plants. Studies have found that the content of ascorbic acid (AsA) and GSH, the ratio of GSSG, the activity of ascorbic acid peroxidase (APX), glutathione reductase (GR) and CAT increased. The levels of H_2_O_2_ and MDA decreased in the salt-pretreated mung beans with proline [59]. Therefore, with the increase in drug exposure time, to resist drug stress, proline content in *Navicula* gradually increased, which might be due to the increase in ROS in algae cells, which stimulated the increase in proline content. Some of them are involved in the osmotic pressure mechanism, acting as protectors of membranes and enzymes, and some are involved in the antioxidant defense system, indirectly scavenging ROS and maintaining the normal growth of algal cells.

Proteins play an important role as functional and structural substances in the plant life process, and different environments or environmental stresses can have specific effects on protein metabolism [60]. Soluble proteins can reflect the active degree of internal plant metabolism and play an essential role in osmoregulation. The amount of their content can reflect the ratio of intracellular protein synthesis and degradation, whether they are denatured or not, and other aspects of information. Under adverse stress, intracellular metabolism cannot be carried out normally, protein synthesis is inhibited, and a stress response is generated in the plant to synthesize new proteins, thus stabilizing intracellular homeostasis [61]. At the same time, soluble proteins are also involved in various metabolic activities in algae, so the change in their content can directly reflect the metabolic situation in algae. When the *Navicula* were subjected to drug stress, it may be because the algal cells changed and turned off some commonly expressed genes to resist the stressful effects of the drug, promptly initiated some genes compatible with the adversity, and synthesized some new antioxidant enzymes or more stable and functional isozymes to compensate for the antioxidant enzymes damaged by free radicals. The gene expression resulted in an increase in protein content. As the exposure time increased, the protein in the algae in G1 and G2 decreased, and the soluble protein content showed a turning point. This phenomenon may be because the antibiotics were too toxic to the *Navicula*. A large number of reactive oxygen species was produced in the algae, which broke the organism’s homeostasis and affected the protein’s structure and composition, exceeding the tolerance of the *Navicula* to ERY and LEV, and hindering the soluble protein anabolism, resulting in protein degradation.

## 5. Conclusions

Briefly, ERY and LEV inhibit algae growth by inducing oxidative damage, inhibiting photosynthesis, and destroying various metabolic activities, thus causing toxic effects on *Navicula*. This study showed that if the amount of antibiotics is not controlled/treated in the water environment promptly, it will pose a potential risk to the aquatic ecosystem in its future use.

## 6. Environmental Implication

The type and concentration of antibiotics in the water environment are gradually increasing, and their potential ecological impact cannot be ignored. As a significant primary producer widely distributed in water bodies, algae are also an essential part of the food chain in aquatic ecosystems. The toxicological effects of pollutants on algae can reveal their threat to algae communities and clarify their potential harm to higher trophic organisms. This study demonstrated the toxic mechanism of high-concentration ERY and LEV and their combined action on *Navicula* as well as their potential damage to the environment, providing a basis for the future biological detection of antibiotics, monitoring and protection of the water environment and rational use of antibiotics.

## Figures and Tables

**Table 1:** Physico-characteristics of erythromycin and levofloxacin.

Compound	Molecular Formula	Molecular Structure	Molecular Weight (g/mol)
Erythromycin	C_37_H_67_NO_13_	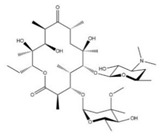	733.94
Levofloxacin	C_18_H_20_FN_3_O_4_	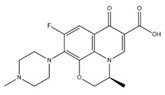	361.37

## Data Availability

No data were used for the research described in the article.
